# A Combined Sensor Design Applied to Large-Scale Measurement Systems

**DOI:** 10.3390/s24175848

**Published:** 2024-09-09

**Authors:** Xiao Pan, Huashuai Ren, Fei Liu, Jiapei Li, Pengfei Cheng, Zhongwen Deng

**Affiliations:** 1 Xi’an Aerocomm Measurement and Control Technology Co., Ltd., Xi’an 710065, China; 2 School of Automation and Information Engineering, Xi’an University of Technology, Xi’an 710048, China; 3 Academy of Advanced Interdisciplinary Research, Xidian University, Xi’an 710126, China

**Keywords:** large-space measurement system, combined sensor, multi-node fusion, signal conditioning, precision measurement

## Abstract

The photoelectric sensing unit in a large-space measurement system primarily determines the measurement accuracy of the system. Aiming to resolve the problem whereby existing sensing units have difficulty accurately measuring the hidden points and free-form surfaces in large components, in this study, we designed a multi-node fusion of a combined sensor. Firstly, a multi-node fusion hidden-point measurement model and a solution model are established, and the measurement results converge after the number of nodes is simulated to be nine. Secondly, an adaptive front-end photoelectric conditioning circuit, including signal amplification, filtering, and adjustable level is designed, and the accuracy of the circuit function is verified. Then, a nonlinear least-squares calibration method is proposed by combining the constraints of the multi-position vector cones, and the internal parameters of the probe, in relation to the various detection nodes, are calibrated. Finally, a distributed system and laser tracking system are introduced to establish a fusion experimental validation platform, and the results show that the standard deviation and accuracy of the three-axis measurement of the test point of the combined sensor in the measurement area of 7000 mm × 7000 mm × 3000 mm are better than 0.026 mm and 0.24 mm, respectively, and the accuracy of the length measurement is within 0.28 mm. Further, the measurement accuracy of the hidden point of the aircraft hood and the free-form surface is better than 0.26 mm, which can meet most of the industrial measurement needs and expand the application field of large-space measurement systems.

## 1. Introduction

The proposed large-space measurement system integrates GPS principles and intersection measurement principles, utilizing 360° laser scanning to construct a non-contact precision measurement system [1,2]. By increasing or decreasing the number of measurement units, the proposed system can balance the limiting relationship between accuracy, efficiency, and measurement area and has the advantages of flexible layout, high measurement accuracy, scalable measurement range, and the ability to carry out parallel measurement of multiple targets in space, which can be applied to the assembly process of aircraft fuselage assembly, shipbuilding, bridge docking, and other large-scale part and component assembly processes [3,4,5]. As the front-end laser signal-receiving device in the system, the photoelectric sensing unit is responsible for realizing the conversion of laser information into digital information that can be collected and calculated, and at the same time, the sensing unit needs to be applicable to a variety of complex measurement areas, which will directly affect the final solution accuracy and measurement range of the system [6,7]. However, existing sensing units can only achieve optical center positioning of the photosensitive surface and cannot directly measure contact points. They also have high requirements for laser signals, making them unsuitable for use in concealed points, such as grooves and cavities, or areas with light obstruction, thus limiting the application of such systems in measurement fields [8].

In response to various measurement scenarios, several different types of photoelectric sensing units have been developed, including planar, spherical, omnidirectional, and combined sensors [9]. Although planar sensors have high measurement accuracy, they are limited by their measurement viewing angle, which has high limitations in practical applications, and they are currently mainly used as standardized measurement systems to calibrate measurement areas [10]. The spherical sensor is different from the former. Through a unique mechanical structure design that makes the optical center of the sensing surface and the center of the ball overlap, spherical sensors can be interchangeable with laser trackers, total stations, and another single-point tracking systems of the target sphere, achieving multi-system fusion, which is conducive to the expansion of the system’s application areas [11]. The omnidirectional sensor receives full-range 360° laser signals through the structure of the sensing surface of the approximate cylinder, which solves the problem whereby the system is unable to carry out intersection measurement due to the blockage of the laser sector when facing a large-space measurement area [12,13,14].

The above three types of sensors have their measurement points located at fixed positions, making it impossible to directly measure the surface coordinates of the target object. To address this issue, a combined sensor design has been proposed. This design enables large-scale mobile coordinate measurement functionality, providing a portable measurement method for the structural dimensions, positions, and morphology accuracy of large components. The combined sensor primarily utilizes multiple spatially arranged photodiodes for mutual constraint and employs a redundant mechanical structure for combined measurements, achieving the goal of measuring contact points [15]. Currently, companies such as Leicia and Nikon have initiated preliminary research and development on this type of sensor [16,17]. Additionally, a research team from Tianjin University has conducted theoretical research on combined measurement methods and developed a circumferential sensor. However, there are still challenges related to measurement accuracy when this sensor is applied in practice [18].

This paper addresses the challenges of measuring hidden points and complex surfaces in large-scale measurement systems by proposing the design of a combined sensor. An efficient measurement method was developed, and the relationship between the number of measurement nodes and measurement accuracy was analyzed. An optoelectronic conditioning circuit suitable for various application scenarios was designed, and a least-squares calibration method based on multi-position vector cone constraints was proposed. The feasibility and accuracy of the proposed design were validated through an experimental platform.

## 2. Measurement Model

### 2.1. Systematic Measurement Model

**Figure 1:**
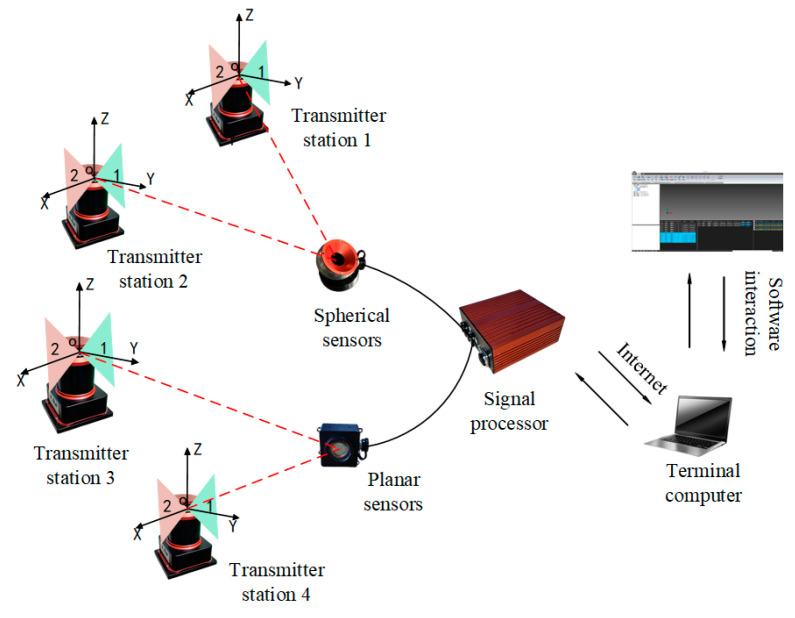
Schematic diagram of the system measurement model.

The large-space measurement system is a typical distributed measurement system primarily composed of laser emission stations, photoelectric sensors, processors, and upper-computer software. As shown in Figure 1, multiple emission stations emit several scanning laser planes within the measurement field which intersect at the photoelectric sensing unit. Subsequent signal processing and computation then enable the three-dimensional coordinate measurement of the target point [19].

In the system, a single transmitter station comprises two laser fan planes oriented at 90° to each other and a rotating axis. At the initial moment, the unit normal vectors of the laser fan planes can be represented by vectors ${\left[\begin{array}{cccc} a_{1} & b_{1} & c_{1} & d_{1} \end{array}\right]}^{T}$ and ${\left[\begin{array}{cccc} a_{2} & b_{2} & c_{2} & d_{2} \end{array}\right]}^{T}$. These parameters remain fixed after the installation of the transmitter station and can be determined through calibration; thus, they are referred to as the internal parameters of the transmitter station.

When the transmitter station rotates and scans the target point, the laser fan plane from the initial moment will have rotated by an angle $\theta_{1}$. The plane equation of the fan can then be obtained by rotating the unit normal vector ${\left[\begin{array}{cccc} a_{1} & b_{1} & c_{1} & d_{1} \end{array}\right]}^{T}$ around the Z-axis, expressed as:

(1) $$\left[\begin{array}{c} a_{1}^{\theta_{1}}\\ b_{1}^{\theta_{1}}\\ c_{1}^{\theta_{1}}\\ d_{1}^{\theta_{1}} \end{array}\right]=\left[\begin{array}{cccc} \cos\theta_{1} & -\sin\theta_{1} & 0 & 0\\ \sin\theta_{1} & \cos\theta_{1} & 0 & 0\\ 0 & 0 & 1 & 0\\ 0 & 0 & 0 & 1 \end{array}\right]\left[\begin{array}{c} a_{1}\\ b_{1}\\ c_{1}\\ d_{1} \end{array}\right]$$

where ${\left[\begin{array}{cccc} a_{1}^{\theta_{1}} & b_{1}^{\theta_{1}} & c_{1}^{\theta_{1}} & d_{1}^{\theta_{1}} \end{array}\right]}^{T}$ denotes the plane equation parameter of sector 1 when it scans over the target point. The plane equation parameter of sector 2 can be obtained as ${\left[\begin{array}{cccc} a_{2}^{\theta_{2}} & b_{2}^{\theta_{2}} & c_{2}^{\theta_{2}} & d_{2}^{\theta_{2}} \end{array}\right]}^{T}$ in the same way.

When the three-dimensional coordinates of the target point under the coordinate system of a single transmitter are ${\left[\begin{array}{ccc} x_{1} & y_{1} & z_{1} \end{array}\right]}^{T}$, and the two laser sectors are scanned over the target point, the three-dimensional coordinates of the target point and the sector plane equations meet the following constraints:

(2) $$\{\begin{array}{c} a_{1}^{\theta_{1}}x+b_{1}^{\theta_{1}}y+c_{1}^{\theta_{1}}z+d_{1}^{\theta_{1}}=0\\ a_{2}^{\theta_{2}}x+b_{2}^{\theta_{2}}y+c_{2}^{\theta_{2}}z+d_{2}^{\theta_{2}}=0 \end{array}$$

According to Equation (2), a single emission station can determine two plane equations. When there are two or more emission stations within the measurement area, the principle of intersection measurement can be applied to achieve a three-dimensional coordinate measurement of the target point.

### 2.2. Combined Measurement Model

**Figure 2:**
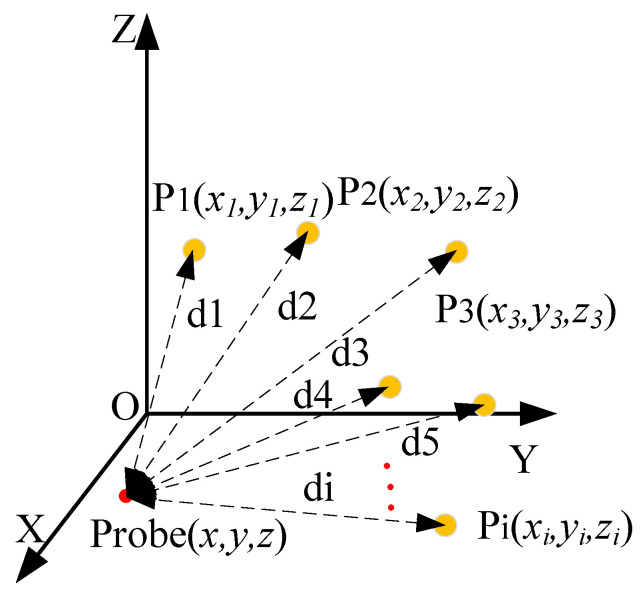
Combined sensor measurement model.

As illustrated in Figure 2, the schematic diagram of the combined measurement model shows multiple sensors arranged on the front-end device. The center of the sensors, denoted as point P, serves as the system’s characteristic point. The distance di between the center of each sensor Pi and the probe point A represents the structural parameters, which can be determined through calibration before measurement [20].

Through the distributed system, the three-dimensional coordinates of each characteristic point on the combined sensor, denoted as ${\left[\begin{array}{ccc} x_{i} & y_{i} & z_{i} \end{array}\right]}^{T}$, can be obtained. By establishing the constraint relationship between each sensor and the probe as expressed in Equation (3), the three-dimensional coordinates of the probe, denoted as ${\left[\begin{array}{ccc} x & y & z \end{array}\right]}^{T}$, can be subsequently determined:

(3) $${\left(x-x_{i}\right)}^{2}+{\left(y-y_{i}\right)}^{2}+{\left(z-z_{i}\right)}^{2}=di^{2}$$

Based on the above equation, it is evident that the combined sensor should contain at least three sensing units. In this study, a redundancy design is adopted, where additional sensors, denoted as n>3, are incorporated. This approach allows for the acquisition of three characteristic point coordinates while enhancing the system’s measurement accuracy through over-determination. Consequently, the system of equations is transformed into an over-determined system, and the optimal solution can be obtained using nonlinear optimization algorithms, as represented in Equation (4):

(4) $$J=\sum_{i=1}^{n}\left[{\left(x-x_{i}\right)}^{2}+{\left(y-y_{i}\right)}^{2}+{\left(z-z_{i}\right)}^{2}-di^{2}\right]$$

where *J* denotes the objective function, and n denotes the sensing unit number on the combined sensor.

## 3. Design of Combined Sensors

### 3.1. Number of Nodes in Simulation

**Figure 3:**
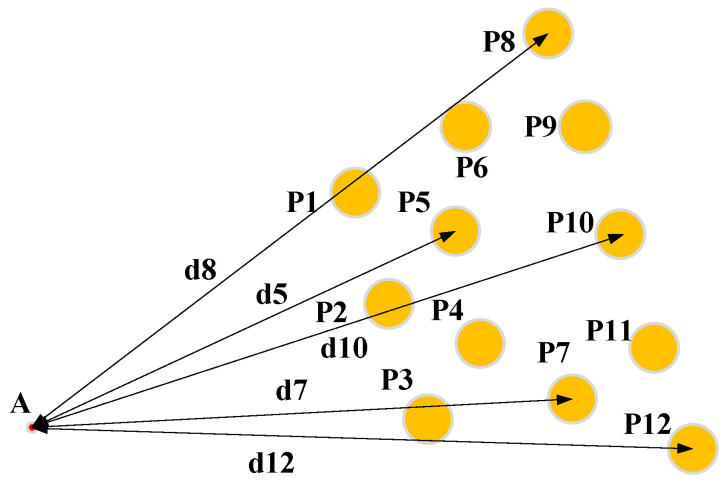
Schematic diagram of combined sensor modeling.

To enhance the measurement accuracy of the combined sensor, the impact of the number of sensing units on the calculation error is investigated. A three-dimensional model is constructed in SolidWorks (Solidworks2020) and combined with MATLAB (MATLAB2019) for simulation analysis, providing a basis for determining the optimal number of nodes in the redundancy design. As illustrated in Figure 3, a measurement model integrating 12 sensing units is shown, where the sensor centers are treated as ideal characteristic points, and the front-end probe A serves as the contact point.

**Figure 4:**
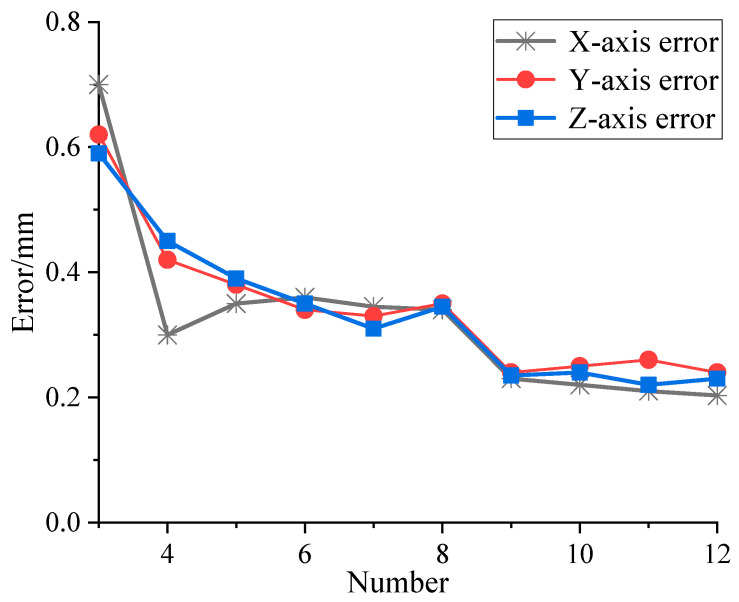
Error curves for different numbers of sensing units.

Combined with the system measurement model and the combined measurement model, the simulation model function is established in MATLAB. The specific simulation steps are as follows:

(1) Extract the coordinates of any three characteristic points and the structural parameters di. To simulate real-world measurement scenarios, a random error within the range of 0.2 mm is introduced to the characteristic points during the simulation. The simulation results are then compared with the theoretical value of point A in the model.
(2) To eliminate the impact of position, a combinatorial approach is employed to repeatedly select three points from the twelve characteristic points for simulation analysis. The absolute error in a single direction is calculated, and the average absolute error is used as the calculation error for the given number of sensing units.
(3) Vary the number of characteristic points and repeat the aforementioned steps to sequentially determine the calculation errors for configurations with three to twelve sensing units. The simulation results are illustrated in Figure 4.

The results demonstrate that as the number of sensors increases, the calculation error gradually decreases, stabilizing after the number of sensing units reaches nine, with an error below 0.23 mm, which satisfies the requirements for large-scale measurements. When simulating four sensing units, a random error of approximately 0.12 mm was observed along the *x*-axis, which is consistent with the random error fluctuations within the system and does not affect the overall trend of the simulation results. Excessive sensing units would increase processor power consumption and computation time; therefore, nine sensing units were ultimately selected as feature points.

### 3.2. Design of Signal Conditioning Circuits

**Figure 5:**
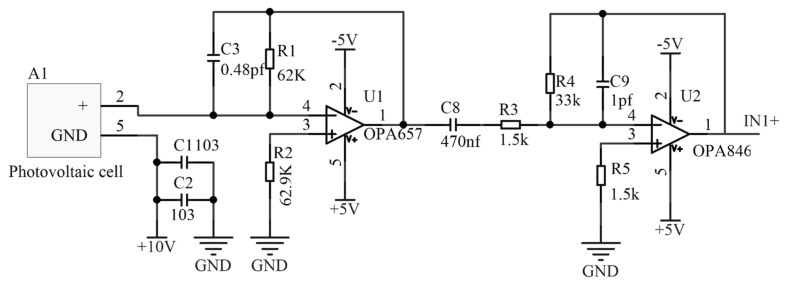
Pre-stage photoelectric conditioning circuit.

The combined sensor, functioning as a handheld device at the front end of the measurement system, requires the conversion of weak laser signals from the transmitter into extractable high-speed, low-frequency pulse signals. To ensure that the current signals output from the photodetectors are accurately converted into voltage signals without distortion, in this study, we designed a wide-bandwidth, low-noise, and high-input-impedance trans-impedance amplifier circuit as the front-end I/V conversion circuit. Additionally, an inverse proportional amplifier circuit is incorporated to enhance the voltage signal strength, as illustrated in Figure 5.

**Figure 6:**
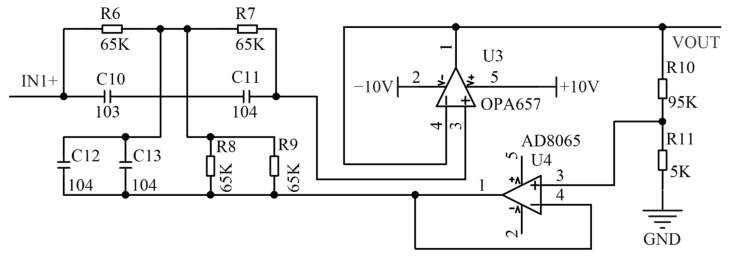
Band-stop filter circuit.

Due to interference from the environment—particularly the significant impact of stray light—on the photodetectors during laser signal reception, in this study, we employed a bandpass optical filter to eliminate such interference. Despite processing, significant periodic baseline noise remains; therefore, a band-stop filter with good frequency attenuation and simple circuitry is designed, as illustrated in Figure 6. This design enables the acquisition of high-quality analog signals.

After the initial signal processing, the signal needs to be converted into a pulse signal for subsequent acquisition. Since the sensor operates in an environment with constantly changing light intensity, the output voltage of the front-end circuit also fluctuates. Therefore, this study employs a hysteresis comparator design that allows for the adjustment of the hysteresis voltage by tuning the adjustable resistor on the SDN port, thereby expanding the operational range of the sensor.

**Figure 7:**
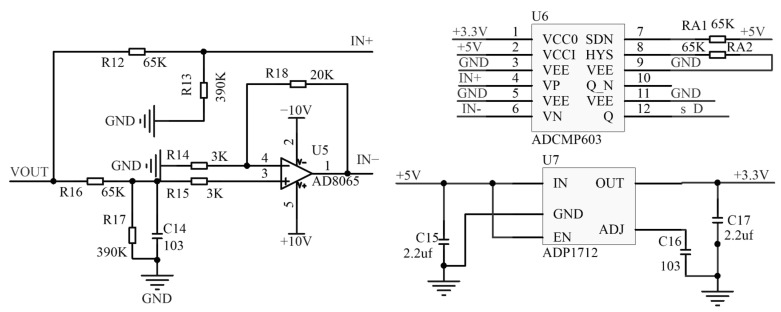
Level comparison circuit.

Additionally, selecting a fixed threshold level may lead to issues such as pulse loss and erroneous output. To address this, an RC integration circuit is designed to filter out the AC components of the signal, while the remaining DC components are proportionally amplified to an appropriate amplitude, as illustrated in Figure 7.

The dynamic threshold level adjusts in response to changes in ambient light, thereby enhancing the signal’s interference resistance and ensuring the accuracy of pulse output.

**Figure 8:**
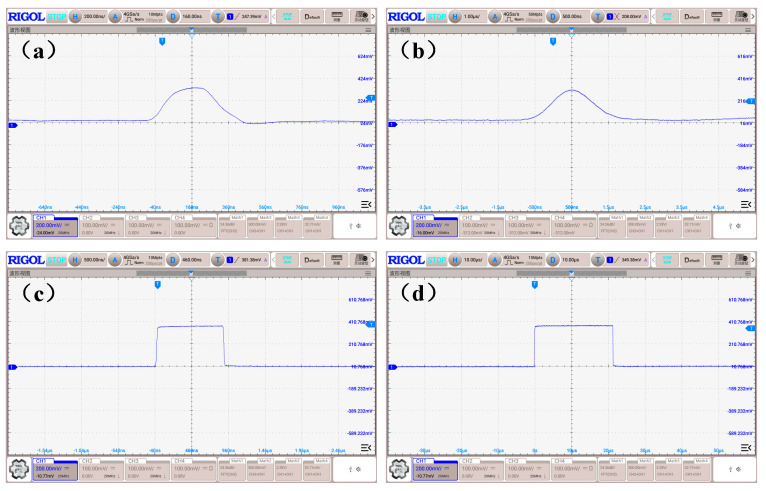
Test waveforms of analog and pulse signals. (**a**) Reference signal. (**b**) Sector signal. (**c**) Reference pulse signal. (**d**) Sector pulse signal.

Subsequently, the functionality of the circuit was validated using a single transmitter station. The waveform outputs of both the analog signal and pulse signal were tested with the detector positioned at the maximum operational distance. As illustrated in Figure 8, the analog signal demonstrated low sensitivity to environmental interference and maintained a high signal-to-noise ratio. The pulse signal exhibited no interference or narrow pulses, with the high-level duration of the fan-shaped laser measuring 20 µs. Moreover, the output waveform displayed good symmetry, effectively enhancing the sensor’s measurement accuracy when integrated with the calculation algorithm.

**Figure 9:**
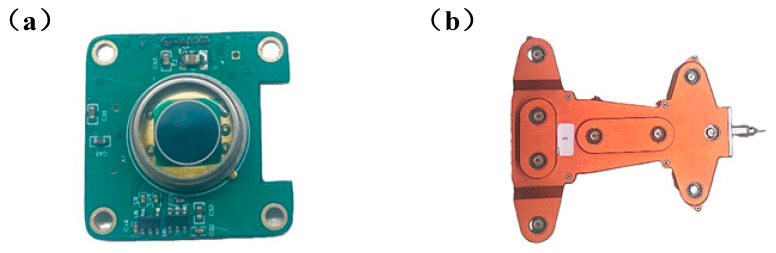
(**a**) Photoelectric conditioning circuit board. (**b**) Physical diagram of the combined sensor.

Following the validation of the circuit’s functionality, the design of the optoelectronic conditioning circuit at the board level was completed. Additionally, the structure of the combined sensor was designed using the number of characteristic nodes, as shown in Figure 9.

### 3.3. Nonlinear Calibration Model

The ideal internal parameters of the combined sensor are the distances from the probe to each feature point. However, due to the inability to ensure processing and assembly precision during actual application, this study employs a high-precision conical calibration platform combined with a distributed measurement system for calibration [21]. By fixing the probe on the calibration platform and keeping the probe position unchanged while altering the feature point positions j times, a set of constraint equations is established, as shown in Equation (5):

(5) $$\{\begin{array}{c} {\left(x-x_{i1}\right)}^{2}+{\left(y-y_{i1}\right)}^{2}+{\left(z-z_{i1}\right)}^{2}=d1^{2}\\ {\left(x-x_{i2}\right)}^{2}+{\left(y-y_{i2}\right)}^{2}+{\left(z-z_{i2}\right)}^{2}=d2^{2}\\ {\left(x-x_{i3}\right)}^{2}+{\left(y-y_{i3}\right)}^{2}+{\left(z-z_{i3}\right)}^{2}=d3^{2}\\ \vdots \\ {\left(x-x_{ij}\right)}^{2}+{\left(y-y_{ij}\right)}^{2}+{\left(z-z_{ij}\right)}^{2}=dj^{2} \end{array}$$

Here, *i* denotes the feature point number, *j* represents the number of times the feature point position changes, and ${\left[\begin{array}{ccc} x & y & z \end{array}\right]}^{T}$ indicates the coordinates of the probe. Similarly, this system of equations is a nonlinear over-determined system, which can be solved using the least-squares method, as shown in Equation (6).

(6) $$v_{ij}^{2}={\left(x-x_{ij}\right)}^{2}+{\left(y-y_{ij}\right)}^{2}+{\left(z-z_{ij}\right)}^{2}-dj^{2},(i=1,2,3,\ldots ,9;j=1,2,3,\ldots ,m)$$

By the node count simulation process described in Section 2.2, the internal parameters of the combined sensor model were extracted, and a simulation model was established. The discrepancies between the simulation results and the ideal internal parameters are detailed in Table 1. The results indicate that after more than six rotations of the sensor, the calibration results converge, with a calibration error of less than 0.018 mm, which meets the measurement requirements for large-scale positioning systems.

## 4. Experiment and Discussion

**Figure 10:**
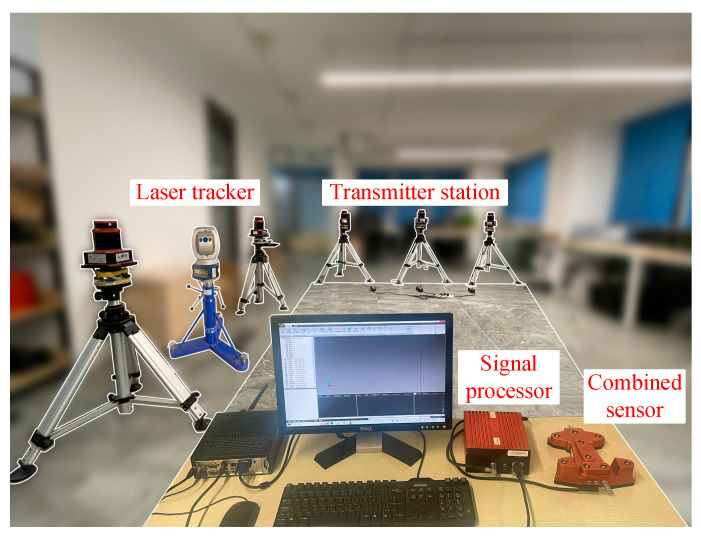
Experimental measurement scenario.

This study utilizes a distributed laser positioning system to validate the accuracy of the combined sensor while incorporating a high-precision laser tracking system as a reference standard (the FARO Vantage laser tracker features a distance measurement accuracy of 16 μm + 0.8 μm/m). Initially, an experimental setup, as depicted in Figure 10, was established within a 7000 mm × 7000 mm × 3000 mm space. The distributed system’s target spheres were designed to be compatible with other systems’ coordinate transformation requirements. Their physical centers are aligned, enabling coordinate unification between the two systems through rotational alignment.

### 4.1. Point-of-Measurement Experiment

**Figure 11:**
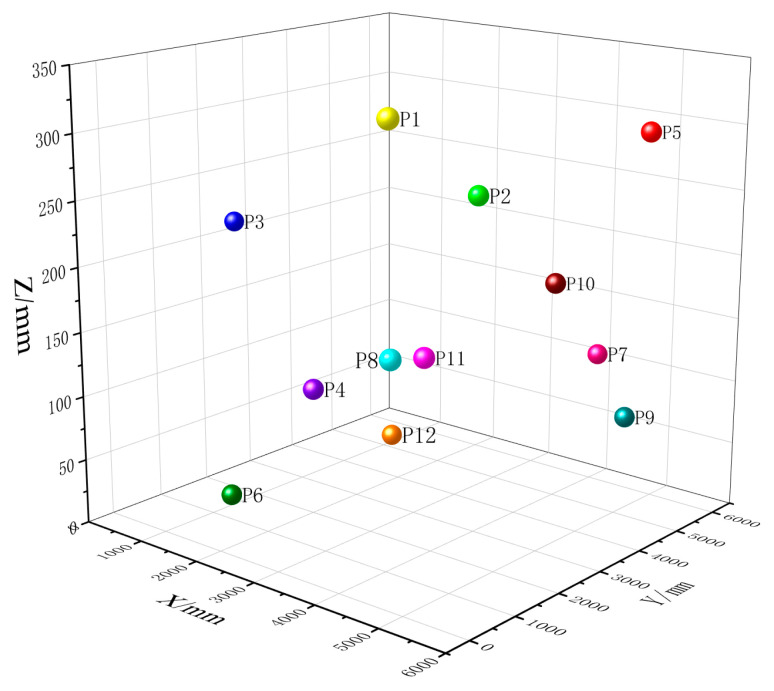
Schematic diagram of measurement points.

**Figure 12:**
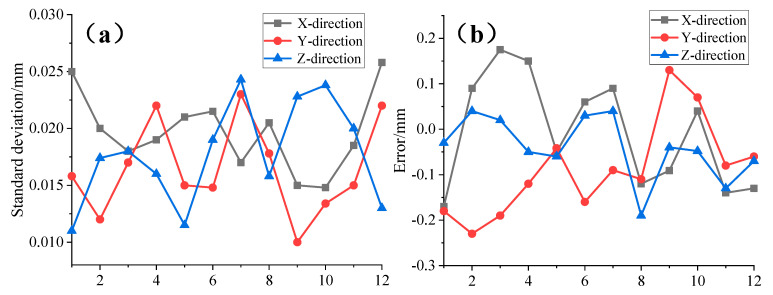
(**a**) The standard deviation of 500 repeated measurements. (**b**) Measurement error compared to the laser tracking system.

Initially, 12 random positions within the measurement area were selected as measurement points, as illustrated in Figure 11. The distributed system performed 500 repeated measurements for each point, and the standard deviation was calculated. Concurrently, the coordinates of these points were measured using the laser tracking system, which served as the reference standard. The deviation of the average of the 500 measurements for each point from this reference was computed. The results are presented in Figure 12.

Experimental results demonstrate that the standard deviations of the combined sensor in the three axes are 0.026 mm, 0.023 mm, and 0.025 mm, respectively. Compared to the laser tracking system, the maximum errors in the three axes are 0.2 mm, 0.24 mm, and 0.19 mm, respectively. These results meet the sub-millimeter precision requirements for large-space measurements and confirm that the proposed design exhibits excellent stability and measurement accuracy.

### 4.2. Distance Measuring Experiment

As the accuracy of measurement points cannot directly assess the distance measurement precision, this section aims to more comprehensively evaluate the measurement accuracy of the combined sensor by verifying its distance measurement capability in practical applications. Unlike the point measurement experiment, this verification will involve fixing a single location and then moving to any 12 locations within the measurement area. The distance measurement results from the laser tracking system will be compared with those from the distributed system, as presented in Table 2.

In this context, position 0 denotes the initially fixed measurement location, while positions 1 through 12 represent twelve randomly chosen locations within the measurement area. The distances *d*1 and *d*2 are calculated relative to the initial position. By comparing the measurement discrepancies between the two systems, the distance measurement error of the combined sensor is determined. The results indicate that the maximum error is less than 0.28 mm, which further validates the reliability of the combined sensor in practical applications.

### 4.3. Functional Experiments

**Figure 13:**
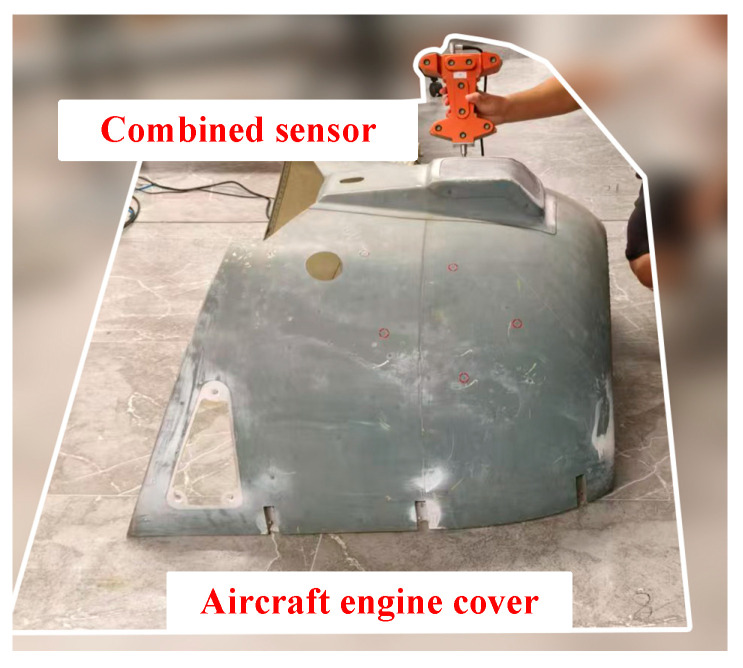
Scenarios of experiments on hidden spots and shaped surfaces of aircraft engine hoods.

After completing the precision testing of the combined sensor, this section evaluates its functionality in detecting hidden points and shaped surfaces using an aircraft engine cover, as shown in Figure 13.

**Figure 14:**
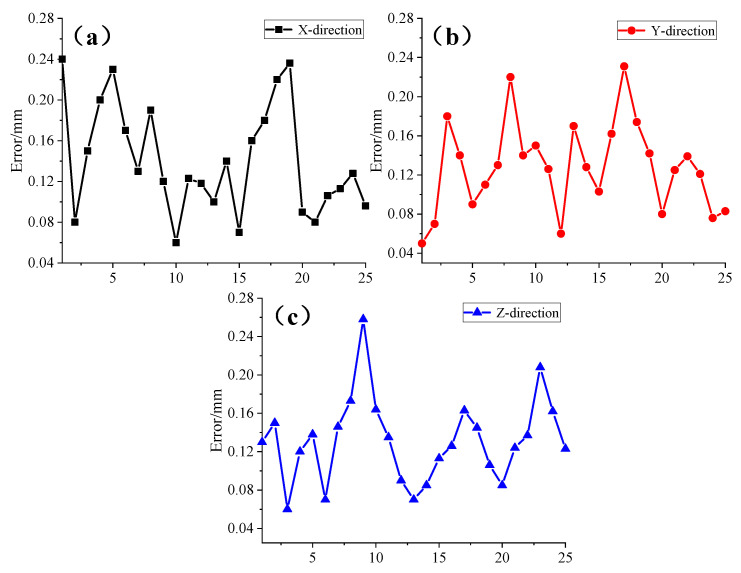
Triaxial measurement error at the hidden point of the airplane hood. (**a**) X-direction. (**b**) Y-direction. (**c**) Z-direction.

The engine cover was placed within the measurement area, and 25 points with optical measurement obstructions or shaped surfaces on the cover were selected. The combined sensor was used to measure the three-dimensional coordinates of these 25 points. Similarly, the laser tracking system was employed to measure the three-dimensional coordinates of these points, and the measurement errors of the two systems were compared. The results, presented as absolute values, are shown in Figure 14.

The experimental results indicate that the combined sensor designed in this study was used to test hidden points and shaped surfaces on an aircraft engine. Compared to the laser tracking system, the maximum coordinate measurement errors in the three axes were within 0.25 mm, 0.24 mm, and 0.26 mm, respectively. Overall, this confirms that the proposed design offers excellent stability and reliability for coordinate measurements, meeting the requirements for most industrial field measurement applications.

### 4.4. Experimental Discussion

Experimental validation demonstrates that the combined sensor designed in this study exhibits excellent measurement stability. Additionally, the integration of absolute precision verification via a laser tracking system confirmed the reliability of measurement points and distance accuracy during application. Testing with an aircraft engine cover has also validated the feasibility of measuring hidden points and shaped surfaces on large components. This further confirms the accuracy of the combined calculation model, feature point quantity, circuit design, and calibration algorithm used in the design process. Compared to the limitations of traditional photoelectric sensors, the combined sensor significantly extends the application range of large-space measurement systems, providing a portable method for measuring structural dimensions, positioning, and the profile accuracy of large components.

## 5. Conclusions

To address the challenges of measuring hidden points and shaped surfaces in large-scale components within large-space measurement systems, this study presents a comprehensive design for a combined sensor. An integrated combined measurement method is proposed which uses constraints based on multiple detection node distances to establish a multi-node fusion model for hidden point measurement and a solution model. The optimal number of nodes is determined through simulation. Additionally, to address challenges associated with the stable conversion of photoelectric signals and environmental adaptability, an adaptive front-end signal conditioning circuit has been developed. A nonlinear least-squares calibration method with multi-position vector cone constraints has also been developed to mitigate the impact of sensors’ internal parameter limitations on measurement accuracy.

By establishing a fusion verification platform with dimensions of 7000 mm × 7000 mm × 3000 mm, the accuracy of the combined sensor was validated. The maximum errors in the three-axis coordinate measurements were found to be within 0.2 mm, 0.24 mm, and 0.19 mm, respectively, while the distance measurement accuracy was within 0.28 mm. Additionally, the coordinate measurements for the aircraft engine cover showed maximum errors of no more than 0.25 mm, 0.24 mm, and 0.26 mm in the three axes. The successful implementation of the combined sensor represents a significant improvement in large-space measurement systems, enhancing their feasibility and stability in various complex measurement scenarios.

## Figures and Tables

**Table 1:** Calibration results of structural parameters.

Number of Revolutions	Calibration Error /mm								
	Δ*d*_1_	Δ*d*_2_	Δ*d*_3_	Δ*d*_4_	Δ*d*_5_	Δ*d*_6_	Δ*d*_7_	Δ*d*_8_	Δ*d*_9_
2	11.457	2.374	0.249	1.328	−9.573	18.375	6.735	4.268	6.747
3	0.027	0.021	0.038	−0.019	0.092	−0.015	−0.044	−0.028	0.024
4	−0.017	0.030	0.078	0.016	0.041	0.029	0.024	0.029	0.017
5	−0.024	−0.135	0.017	0.021	0.064	0.015	0.011	−0.017	−0.009
6	0.009	0.007	−0.005	0.015	−0.018	0.007	0.009	−0.011	0.014
7	0.004	0.012	−0.007	0.008	0.003	0.015	0.009	−0.012	−0.007
8	0.013	0.009	−0.008	−0.012	0.007	0.012	0.0015	−0.013	−0.006
9	0.012	−0.011	0.007	−0.013	−0.007	0.008	0.0013	0.014	−0.009

**Table 2:** Experimental results of combined sensor ranging accuracy.

Number	Distributed System/mm	*d*1/mm	Laser Tracker/mm	*d*2/mm	Error/mm
0	(3274.40, 2260.15, −632.02)	0	(3274.08, 2260.42, −632.19)	0	0
1	(2238.27, 1248.59, −431.21)	1461.9	(2238.61, 1248.24, −430.07)	1462.04	0.14
2	(1316.43, 2758.27, −592.18)	2020.73	(1316.19, 2758.21, −592.27)	2020.57	−0.15
3	(4962.31, 2965.54, 1120.35)	2533.26	(4962.14, 2965.72, 1120.43)	2533.51	0.25
4	(4735.34, 1394.65, 546.31)	2066.86	(4735.21, 1394.8, 546.12)	2067.03	0.17
5	(961.71, 3598.32, 2236.84)	3920.41	(961.42, 3598.5, 2237)	3920.6	0.19
6	(942.68, 1658.43, 1240.52)	3051.09	(942.21, 1658.68, 1240.69)	3050.8	−0.28
7	(3051.37, 66.78, −2481.39)	2878.40	(3051.98, 66.42, −2481.72)	2878.15	−0.25
8	(5962.21, 4748.35, −104.63)	3700.63	(5962.3, 4748, −104.55)	3700.4	−0.23
9	(6333.46, 2102.94, 98.6)	3149.03	(6333.42, 2102.4, 98.05)	3149.25	0.22
10	(3574.61, 2201.3, 748.54)	1414.05	(3574.38, 2201.43, 748.6)	1414.3	0.24
11	(2963.15, 1483.37, 1836.95)	2606.93	(2963.52, 1483.12, 1836.48)	2606.72	−0.21
12	(1752.68, 4658.42, 967.75)	3259.85	(1752.45, 4658.43, 967.5)	3259.57	−0.27

## Data Availability

Data are contained within the article.
